# 674. Association Between Self-reported and Performance-based Physical Function in Burn Survivors: An Ongoing Prospective Study

**DOI:** 10.1093/jbcr/irag033.555

**Published:** 2026-04-06

**Authors:** Julia Kleinhapl, Victoria G Rontoyanni, Carole A Tucker, Steven E Wolf, Oscar E Suman-Vejas

**Affiliations:** Division of Burn and Trauma Surgery, Department of Surgery, University of Texas Medical Branch, Galveston, Galveston, TX; University of Texas Medical Branch, Galveston, TX; University of Texas Medical Branch, Galveston, TX; Division of Burn and Trauma Surgery, Department of Surgery, University of Texas Medical Branch, Galveston, Galveston, TX; University of Texas Medical Branch, Galveston, TX

## Abstract

**Introduction:**

Complications after burns, such as loss of muscle mass and bone density, hinder the regain of physical fitness and rehabilitation. While patient-reported physical function is commonly assessed, its association with objective performance remains underexplored in burn populations. Our objective was to quantify the level of associations between self-reported physical function and performance-based measures of physical function after burns.

**Methods:**

We prospectively assessed physical function of adult burn patients who underwent surgery within 30 days of injury at hospital discharge (±14 days), 3 months (±30 days), and 6 months (±60 days). Self-reported physical function was measured with the Patient-Reported Outcome Measurement Information System (PROMIS)-29 v2.1. T-scores were derived via the HealthMeasures Scoring Service. Objective physical function performance assessments included cardiopulmonary endurance (modified Bruce treadmill protocol) and muscle function by isokinetic dynamometry. Muscle function was indexed by peak torque normalized to body weight (PEAK TQ/BW%, knee extension), cardiopulmonary effort by percent heart-rate reserve (%HRR = (HRpeak-HRrest)/(HRmax,pred-HRrest)x100). Age-predicted maximal heart rate: HRmax,pred = 208 − 0.7xage. Cross-sectional correlations between questionnaire physical function outcomes and performance-based physical function measurements were assessed using one observation per patient with Spearman’s rank correlation (two-tailed; α = 0.05). Demographics are reported as medians and interquartile ranges (IQR). Analyses were performed in GraphPad Prism.

**Results:**

To date, 7 patients completed at least one time point with both self-reported questionnaire and physical-function assessments. The median age was 52 years (IQR 33–58), sex distribution female:male = 1:6, median body mass index (BMI) 29.7 kg/m^2^ (IQR 24–31), and median total body surface area burned 6% (IQR 2–16). We observed a non-significant correlation between the PROMIS-29 Physical Function T-score (same visit) and peak torque (PEAK TQ/BW%): Spearman’s ρ = 0.25, *p*=.595 (n = 7). The correlation between the PROMIS Physical Function T-score and cardiopulmonary effort (%HRR) was also not significant (Spearman’s ρ = 0.43, *p*=.35, n = 7).

**Conclusions:**

In this preliminary sample, PROMIS-29 Physical Function T-scores showed weak, non-significant correlations with performance-based physical function. Though we recognize the small sample, our results suggest limited agreement between subjective and objective assessments of physical function, underscoring the need to obtain both types of assessments for a more comprehensive view of a patient’s physical function and recovery.

**Applicability of Research to Practice:**

Given the lack of a strong correlation in this small cohort, these data support using both standardized objective physical function measures alongside PROMIS to inform and tailor rehabilitative exercise plans.

**Funding for the study:**

Leon Hess Professorship; National Institute on Disability, Independent Living, and Rehabilitation Research (NIDILRR) 90DPBU0003; 90IFRE0079-01-01; Remembering the 15 Endowment Funds.

